# Plant Responses to Climate Change: The Case Study of Betulaceae and Poaceae Pollen Seasons (Northern Italy, Vignola, Emilia-Romagna)

**DOI:** 10.3390/plants5040042

**Published:** 2016-12-06

**Authors:** Anna Maria Mercuri, Paola Torri, Rita Fornaciari, Assunta Florenzano

**Affiliations:** 1 Laboratorio di Palinologia e Paleobotanica, Dipartimento di Scienze della Vita, Università degli Studi di Modena e Reggio Emilia, Viale Caduti in Guerra, 127, 41121 Modena, Italy; annamaria.mercuri@unimore.it (A.M.M.); paola.torri@unimore.it (P.T.); assunta.florenzano@unimore.it (A.F.); 2 Plant Physiology Lab, Dipartimento di Scienze della Vita, Università degli Studi di Modena e Reggio Emilia, Via Kennedy, 17/I, 42124 Reggio Emilia, Italy

**Keywords:** pollen, aerobiology, global warming, wind-pollinated plants, long-term series, *Betula*, *Alnus*, *Corylus*

## Abstract

Aerobiological data have especially demonstrated that there is correlation between climate warming and the pollination season of plants. This paper focuses on airborne pollen monitoring of Betulaceae and Poaceae, two of the main plant groups with anemophilous pollen and allergenic proprieties in Northern Italy. The aim is to investigate plant responses to temperature variations by considering long-term pollen series. The 15-year aerobiological analysis is reported from the monitoring station of Vignola (located near Modena, in the Emilia-Romagna region) that had operated in the years 1990–2004 with a Hirst spore trap. The Yearly Pollen Index calculated for these two botanical families has shown contrasting trends in pollen production and release. These trends were well identifiable but fairly variable, depending on both meteorological variables and anthropogenic causes. Based on recent reference literature, we considered that some oscillations in pollen concentration could have been a main effect of temperature variability reflecting global warming. The duration of pollen seasons of Betulaceae and Poaceae, depending on the different species included in each family, has not unequivocally been determined. Phenological responses were particularly evident in *Alnus* and especially in *Corylus* as a general moving up of the end of pollination. The study shows that these trees can be affected by global warming more than other, more tolerant, plants. The research can be a contribution to the understanding of phenological plant responses to climate change and suggests that alder and hazelnut trees have to be taken into high consideration as sensible markers of plant responses to climate change.

## 1. Introduction

Plants adopt many strategies to face climate changes. There are strategies including shifting of vegetation at association scale, or strategies raising the rate of physiological processes such as photosynthesis at the community and individual scales. In fragmented landscapes such as urban habitats, current rapid climate change has the potential to overcome the capacity for adaptation in plant species, also altering genetic composition and biodiversity [1].

Phenology shows evident responses to long-term variations of temperatures (e.g., [2]). The male gametophytic phase has proven to be one of the most temperature-vulnerable stages, often highly sensitive to hot or cold stresses on a short-term basis. Pollen germination and pollen tube growth show acceleration under increasing temperatures (in peach: [3]) or, contrarily, can be negatively affected by heat [4]. Among the dramatic climate changes, the recent trend towards global warming is overall accepted, and an increase of about 0.6 degrees on mean temperatures since the late 19th century has been acknowledged [5]. In general, pollen development and fertilization may be the most sensitive reproductive stage to temperature increase and, therefore, the current global warming is recognized as having clear effects on pollen production and fertility.

Aerobiological data have especially demonstrated the correlation between climate warming and pollination seasons [6,7,8,9,10,11]. Following this approach, we report on the trend of airborne pollen data from two of the most important botanical families at mid latitudes, Betulaceae and Poaceae. In European countries, they represent large part of the current plant cover as woody (Birch family) and herbaceous (grasses) species. Both the Birch family (*Alnus*, *Betula*, *Carpinus*, *Corylus*, *Ostrya*) and grasses (wild and cultivated, including cereals) are wind-pollinated and produce among the vast amounts of pollen in the spring and summer airflows in Europe [12]. Despite cleistogamous species obviously making a light contribution to airborne pollen rain, grasses are responsible for the most important allergy symptoms registered in the world (e.g., [13]).

This paper focuses on temperature as a major factor influencing the pollen season with respect to precipitation [14,15]. Precipitation, wind speed, and wind direction have an important role in the spreading and amount (concentration) of pollen in the air. However, temperatures higher than normal are able to improve pollen quantity and diffusion, frequently resulting in longer seasons of pollination in many plant species ([16,17,18,19]; see [20] for grasses). In this paper, we focus on this aspect and observe the trend of a long series of airborne pollen monitoring by considering the possible correspondence between pollen concentrations and variation of temperatures.

The paper reports on data collected from one of the first airborne monitoring station located in Italy (at Vignola, MO2). The monitoring station started running in 1989. It has produced only fragmented data in recent years, and it is not working anymore. Therefore, we selected the long interval 1990–2004 during which data had been collected on a continuous basis and for the whole year. The 15-year set of data represents a long series offering the possibility to compare pollen trends and temperatures over a relatively long timespan. Other examples of long series published in the last couple of years are: 16-year period (1994–2009) to predict olive crop production [21]; 15-year period (1993–2007, coupled with another 1992–2011) to model and predict the dynamic of *Vitis* flowering in the field [22]; an 11-year study (2003–2013) to quantify the effects of environmental factors including pollen on asthma hospitalization [23]; a 30-year series (1981–2010) is useful to compare historical data with atmospheric dispersion model for airborne pathogen concentrations [24].

## 2. Results

**Figure 1:**
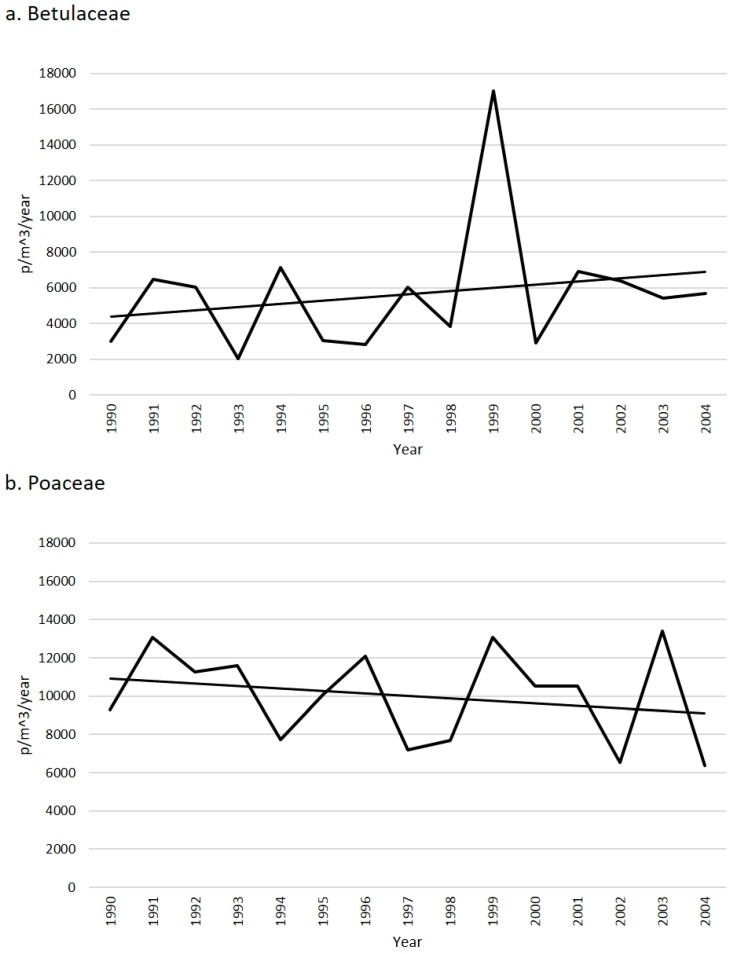
Yearly Pollen Index (YPI) of Betulaceae (**a**) and Poaceae (**b**) from 1990 to 2004.

The trends of the Yearly Pollen Index (YPI) of airborne pollen spread of the two families from 1990–2004 show comparable and variable concentrations, with general contrasting patterns (Figure 1). Below, the general data are reported aiming at introducing the main features of the concentration variability that has been observed in the pollen rain produced by these anemophilous pollen grains in the studied area. They may be paradigmatic of pollen concentration trends under changing weather conditions over 15 years.

### 2.1. Betulaceae

**Figure 2:**
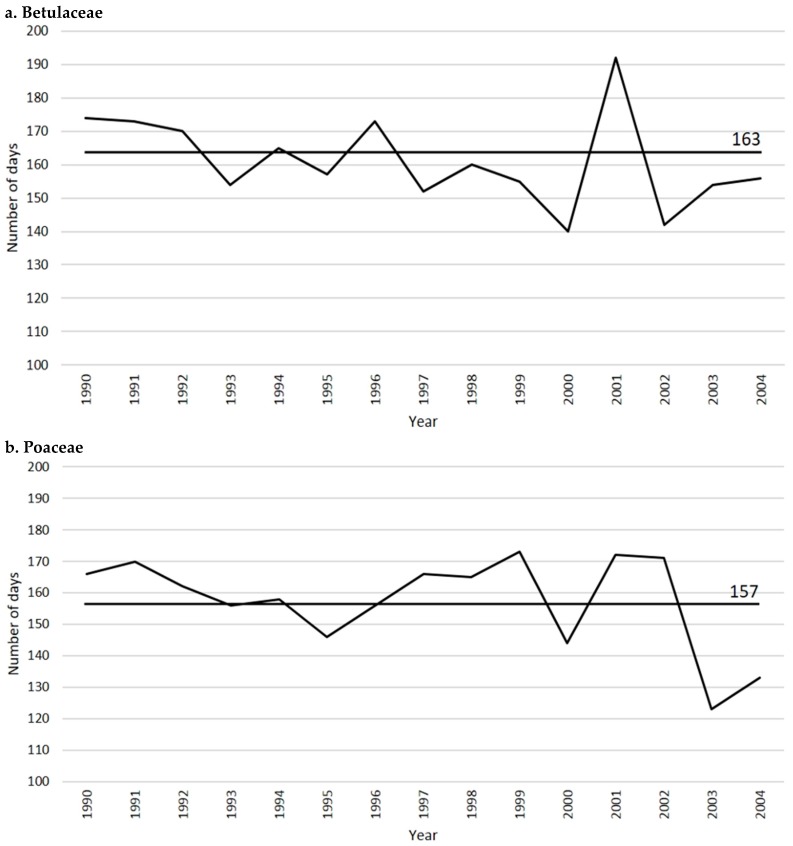
Duration of pollen season of Betulaceae (**a**) and Poaceae (**b**) from 1990 to 2004.

The pollen season is 163 days per year on average. The longest season occurred in 2001 (192 days, mainly due to *Alnus*) whilst the shortest season occurred in 2000 (140 days; Figure 2a). The highest YPI (Figure 1a) occurred in 1999 (17,012 p/m^3^): this value was obtained from the very high values of *Ostrya* pollen concentration (12,653 p/m^3^) followed by significant values of *Alnus* (1869 p/m^3^) and *Corylus* (1656 p/m^3^).

**Figure 3:**
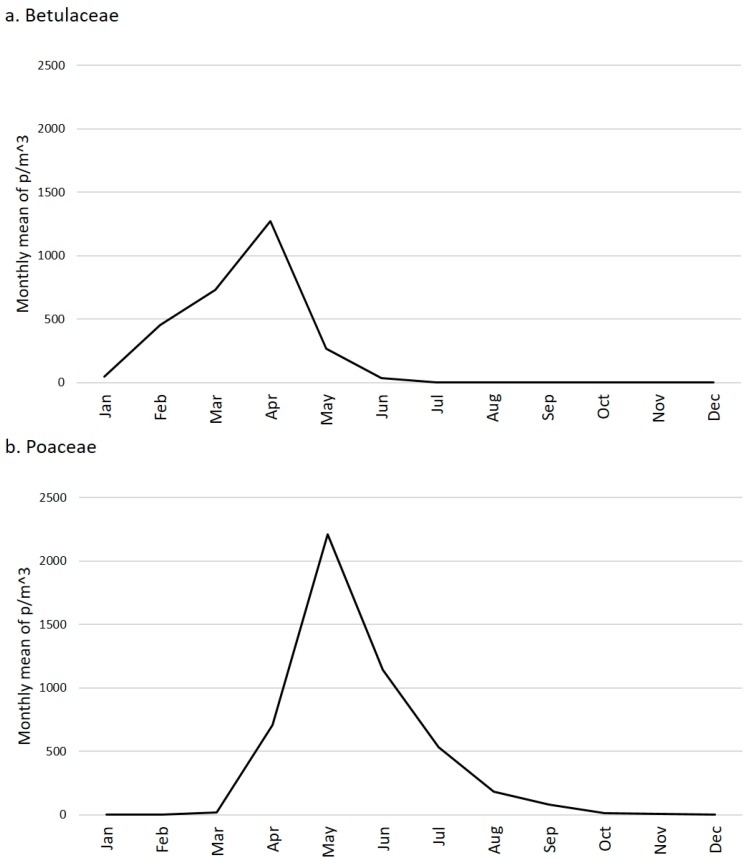
Months composing the pollen season of Betulaceae (**a**) and Poaceae (**b**): total monthly mean of each year from 1990 to 2004.

The months composing the pollen season were from January to late July (Figure 3a). Depending on the different plant genera/species included in the family, the pollen seasons started in January (until May for *Corylus*, July for *Alnus*) and February (until June for *Betula* and *Ostrya*, May for *Carpinus*).

**Figure 4:**
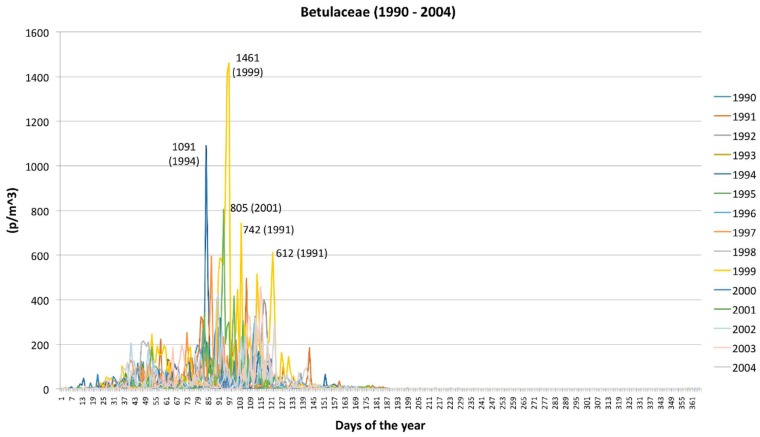
Daily peaks of pollen concentration of Betulaceae: a comparison from 1990 to 2004.

The daily peaks of pollen concentration were observed in the years 1994 (1100 p/m^3^ in March), 1999 (1460 p/m^3^ in April), and in 2001 (800 p/m^3^ in April) whilst a very low maximum value (<150 p/m^3^) was observed in 1993 and 2000 (Figure 4). In the other years, the daily peaks were about 200–600 p/m^3^ and, in general, were reached in April. In all years, the pollen shows a decreasing trend in summer with some re-increase of concentration in June due to the blooming of *Alnus viridis* (especially visible in 1994 (see also [25])).

The end of the pollen season of this family has been actually recorded at this time in the considered period.

### 2.2. Poaceae

The pollen season consists of >100 days per year (157 days on average). The longest season occurred in 1999 (173 days) whilst the shortest season occurred in 2003 (123 days; Figure 2b). Accordingly, the highest YPI (Figure 1b) occurred in the years with the longest pollen season (1991, besides 1999).

The months composing the pollen season were from late March to early September (Figure 3b). In 1994, most of the pollen concentration was especially concentrated at the early period of the season (late March), and in general pollination started in this period during other three years (1997, 2001, 2002). In the other years, the first half of April saw the start of the pollen season.

**Figure 5:**
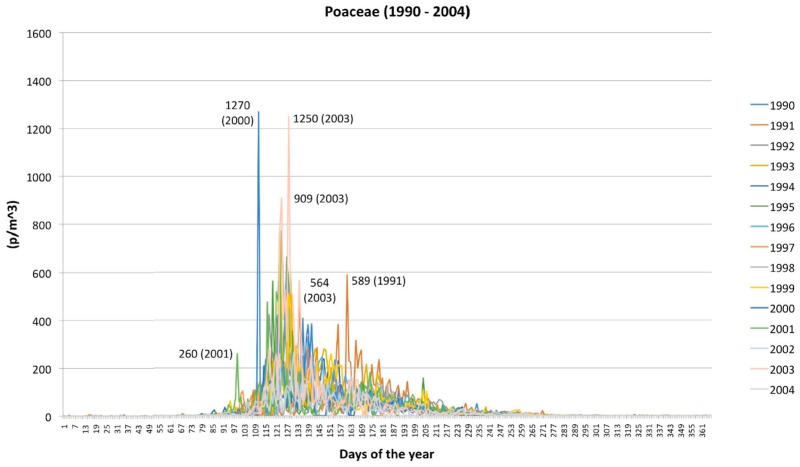
Daily peaks of pollen concentrations of Poaceae: a comparison from 1990 to 2004.

The daily peaks of pollen concentration (>1200 p/m^3^) were observed in the years 2000 (in April) and 2003 (in May) whilst low maximum values (<300 p/m^3^) were observed in 1994, 1997, 2002, and 2004 (Figure 5). In the other years, the daily peaks were 600 p/m^3^ and, in general, were reached in May. The years 1991 and 1994 are exceptions as the daily maximum value was reached in June. In all years, the pollen shows a decreasing trend in summer with some re-increase of concentration at the end of July (in 1995 and 1999), or at the end of August (in 1990).

The end of the pollen season has been actually recorded at this time in a few years (1995, 2000, 2001, 2003) whilst it occurred in September in many years of the considered period.

## 3. Discussion

Aerobiological data can explain unpredictable annual variability of concentrations over the years but cannot be fully responsible for the increasing trend of Betulaceae (Figure 1a) versus the decreasing trend of Poaceae (Figure 1b). In general, the monthly trends and concentrations follow patterns that are typical and repeated after a seasonal basis. Thus, the Betulaceae pollen season is January–July with acme in April whilst the Poaceae pollen season is March–September with acme in May (Figure 3). Data are in agreement with the general information that is usually obtained from the European pollen monitoring network [26].

Aerobiological data from Betulaceae and Poaceae show that pollen seasons depend on the phenology of the different species included in each family. On one hand, human interference with flowering cycles or plant distribution and growing are common as cutting of trees, mowing of herbs, and garden or park maintenance in urban habitats. They can be responsible for unpredictable anomalies in airborne pollen concentrations. On the other hand, a high number of species is included in these families, obviously influencing trends and the variability of pollen production. The genera and species have variable blooming seasons. For example, among Betulaceae, there are species of *Alnus* blooming in April and others in July–August. In the study region, during the spring period in March–April, spore traps can capture alder airborne pollen from *Alnus cordata* (Loisel.) Duby, Italian Alder; *A. glutinosa* (L.) Gaertn., European Alder; and *A. incana* (L.) Moench, Gray Alder. The latter species blooms until May. Data from the monitoring station of Vignola, here discussed, also systematically showed alder pollen in the air in July and August; this is clearly related to another species, *A. viridis* (Chaix) DC that does not live in Emilia Romagna. The Green Alder is known to be distributed in Northern Italy, on mountains from the West Piedmont to the East Friuli. This means that its pollen has arrived, and still arrives, from the northern Alps as a long-distance transport with summer air masses [25,27].

**Figure 6:**
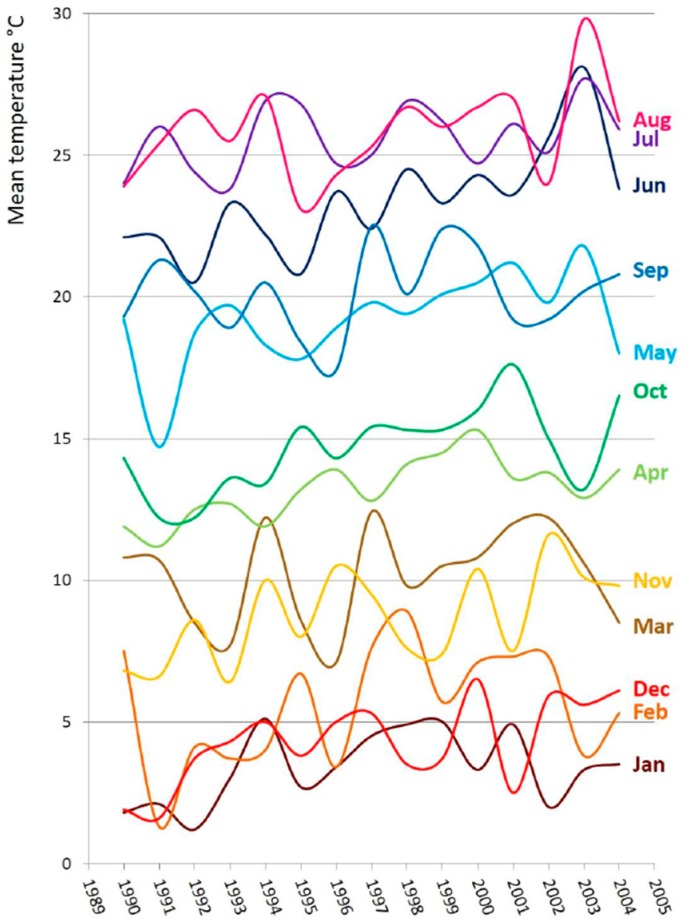
Monthly mean temperature during the considered period (1990–2004).

Poaceae is a large group including species with different flowering periods, and very different pollen production [28], which may result in more than one peak during the grass pollen season. Moreover, there are grasses with long periods of pollination or blooming more than once in the year: e.g., *Poa pratensis* L., smooth or common meadow-grass, from May to July; *Lolium perenne* L., perennial ryegrass, and *Cynodon dactylon* (L.) Pers., Bermuda grass, from May to September. According to their nature of herbaceous plants, frequently owing a short-life cycle and growth limits due to the anthropogenic control, Poaceae show more evident (casual) oscillations in the YPI than Betulaceae. Interestingly, the maxima daily concentrations (>1200 p/m^3^) of Poaceae were observed in the years 2000 and 2003 (Figure 5) that are among the warmest years of the last century in the considered period (see especially summer temperatures in Figure 6). In particular, according to a survey by the National Environmental Agency, mean temperatures of the same years had an increase up to +1.38 °C in Italy. Moreover, the combination of the highest YPI and the shortest pollen season of Poaceae occurred in 2003. By comparing the analyses from several towns located in Spain [29], some similarities are evident: oscillations of pollen concentration do not follow a clear trend, pollen season never exceeds 200 days, and the maximum value of concentration was reached in 2003.

In general, these data support the idea that Poaceae replies to temperature increase with a higher production of pollen, eventually resulting in high concentration for a short period.

Poaceae data show that the duration of the pollen season influences the YPI. For example, in the years 1991 and 1999 there were high values of grass pollen concentration and long pollen seasons (Figure 2b). In 1999, an anomalous (high) value of the YPI of Betulaceae was also registered, especially due to *Ostrya*, and partly also due to *Alnus* and *Corylus*.

**Figure 7:**
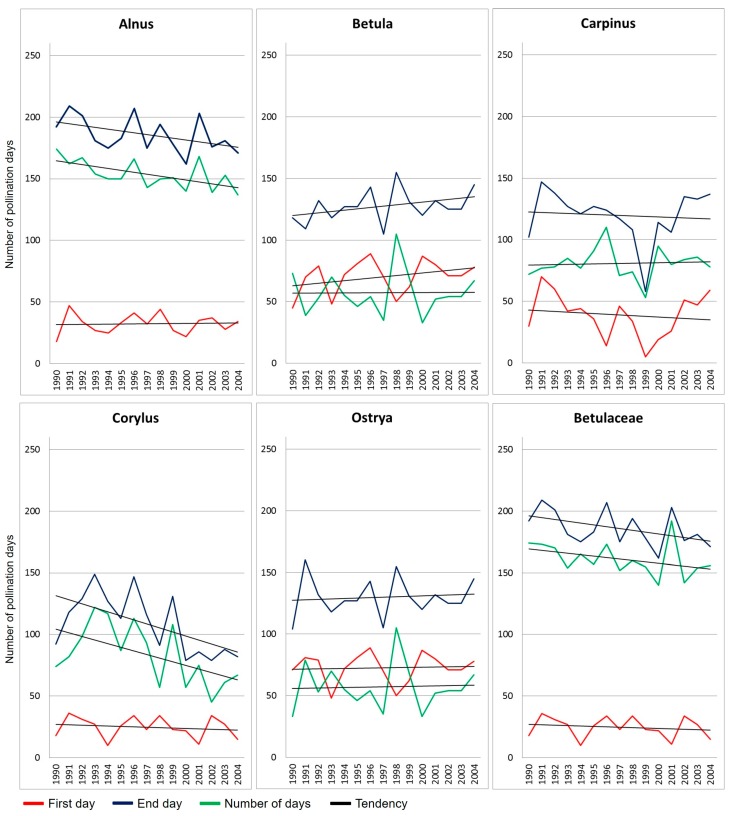
Pollen season of the five genera in the Birch family and in total Betulaceae, from 1990 to 2004.

In general, the pollen season of Betulaceae ranges between 140 and 200 days in different years. The Birch family tends to decrease the number of days with pollen in the air but important distinctions were observed among the genera. *Ostrya* and *Carpinus* had a quite steady duration of the pollen season, while the number of days with pollen tends to increase in *Betula* and to decrease in *Alnus* and *Corylus* (Figure 7). For example, there is a slight increasing trend of *Betula* from 56 to 58 days with pollen in the air, passing from the 1990–1995 interval to the 1996–2004 interval. The duration of the pollen season of *Alnus* decreases from 160 in 1990–1995 to 150 days in 1996–2004. *Corylus* pollen season ranges as mean from 97 days in 1990–1995 to 75 days in the years 1996–2004. Interestingly, there are signs that a similar behavior characterized the way the pollen seasons changed. All the genera show a tendency to maintain almost steady pollination in the early days of the pollination season all over the years. Conversely, the end days show important differences: they pass from a delay of about 2 days for *Betula* (corresponding to an increase of 3.5% of the number of days with *Betula* pollen in the air), to a reduction of about 10 days in *Alnus* (decrease of 6.5%), and of about 22 days in *Corylus* (decrease of 25.6%; Figure 7).

Despite the obvious observation that airborne pollen concentration for all species decreases with precipitation, and pollen concentration is low/insignificant in a day with rain or the day after (e.g., [30]), most research points to the main significance of temperature for pollination. An 18 year long-term study (1989–2006) on the characterization of the male flowering and pollen flow of birch in Finland-northern Europe was carried out to understand the proximal causes contributing to pollination, and the annual variability and spatial synchronization of male catkin numbers of silver birch (*Betula pendula* Roth.) and downy birch (*Betula pubescens* Ehrn.). The research demonstrated that there is no correlation between catkin numbers, annual airborne pollen sums, and the amount of precipitation during the pollination season [31]. Indeed, studies report on the correlation between temperatures and pollen seasons where different tree species give different responses to climate change. The focus has commonly been directed to the start of pollination.

Studying the warming conditions of the last years, from monitoring stations of Northern Europe it emerges that *Alnus*, *Betula*, and *Corylus* generally have an early pollination start. As *Betula* lives at low temperatures in mid to high latitude countries, data from Central Europe suggest that an increase in temperature could limit the physiological performance of birch species, including the production of pollen [32]. European data, however, reveal that *Alnus*, *Betula*, and *Corylus* trees are more associated with high latitudes and low temperatures than others, and their annual concentrations have a negative correlation with temperature that could reflect the limited presence of these species at warmer sites [33]. In Italy, moving to different local geobotanical features, in the last years the pollination start tends to become delayed in winter-blooming plants (*Alnus*, *Corylus*) while the most important tendency to an early pollination occurs in spring-blooming trees (*Betula*) [6,19]. Our data add information about the behavior of the last day of pollination periods. Pollen monitoring suggests that there is a tendency of *Alnus* and *Corylus* to behave as winter-blooming trees thus anticipating the end (earlier last day) of their pollen seasons. Contrarily, the spring-blooming *Betula* trees tend to delay the end of the pollen season. Comparing these results, we can conclude that the pollen season of alder and hazelnut trees tends to be shorter, meanwhile the season of birch becomes longer. This seems to demonstrate that warm climate, as the current environmental conditions are, may be generally unfavorable to the winter-blooming trees that have a later pollination after exceptionally warm autumn temperatures [27].

## 4. Materials and Methods

**Figure 8:**
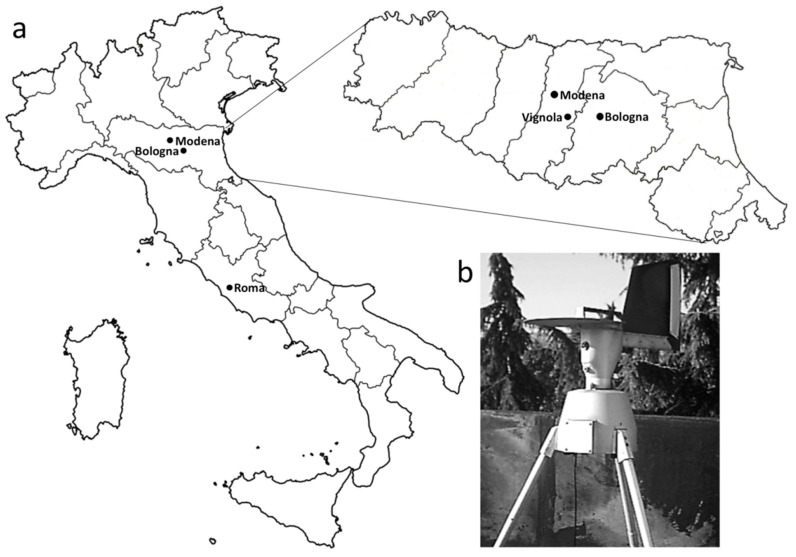
(**a**) Location map of Vignola, Emilia-Romagna, Northern Italy; (**b**) Lanzoni VPPS 2000 spore trap.

The pollen monitoring station was located in Vignola (MO2; 125 m a.s.l.; 44°29′ N 11°00′ E), a small town at the center of Emilia-Romagna, in Northern Italy. The region includes the Po plain, the Tuscan-Emilian Apennines to the south, and the Adriatic Sea coast to the east (Figure 8). The Po plain has a climate gradient ranging from the Mediterranean warm-temperate to the cold-temperate climate of the Apennines. Based on the mean temperature of the coldest month (January; from −4 °C to +4 °C), Emilia-Romagna falls in the temperate (C) and warm-temperate (D) thermic zones by Pignatti [34].

The airborne pollen monitoring station was part of both the Italian and the European A.I.A.—Aeroallergen monitoring networks. The station was in continuous operation from 1990 to 2004. A seven-day Lanzoni VPPS 2000 spore trap (a Hirst volumetric sampler; Figure 8) was located at approximately 12 m on the roof of the Local Health Agency. Sampling and slide mounting followed standard methods of the aerobiological networks [35,36,37]. The daily slides were analysed with a light microscope at 250× magnification (400–1000× for details), and pollen grains were counted along four longitudinal transects. Pollen identification was done through current pollen atlases and keys and the reference pollen collection. Pollen data were elaborated using the softwares GEPO and “Aerobiologia 2.1” [38].

The Yearly Pollen Index (YPI) is the total amount of one pollen type in one year and it is given by the sum of the daily mean concentrations. The pollination period corresponds to the days between start and end days of pollen in the air, and refers to the continuous presence of the studied pollen in the air. The start of pollination was established as the first time that the pollen grains of the studied taxon were observed in slides from three subsequent days [9,39,40]. The pollination period considered for each specific pollen taxon include >90% of its yearly total pollen.

Data were imported into Microsoft Excel 2013 and used for building the graphs. The linear trend was calculated by the coefficient of determination (*R*^2^) using linear analysis tools.

Temperature and precipitation data were available from one meteorological station of Modena (e.g., [41]) and from the Regional Agency for the Protection of the Environment (A.R.P.A.).

## 5. Conclusions

The 15-year aerobiological data analysis reported from the monitoring station of Vignola shows that trends in pollen production and release are easily identifiable but fairly variable, depending on meteorological variables besides anthropogenic causes. Despite data collection being interrupted about 12 years ago, the interest of these results is still effective as they provide an opportunity to observe phenological responses to temperature changes that had occurred in the considered years, under increasing mean temperature values.

The pollen season of Betulaceae, like most of the tree families at middle latitudes, covers the first part of the year, from winter to early summer. The pollen season of Poaceae starts later, principally covering the spring and summer seasons. In our data, the highest YPI and the shortest pollen season of Poaceae occurred in 2003 suggesting that, in general, these phenological events match the presence of average warm temperatures. Poaceae perform a higher pollen production (i.e., high pollen concentration in the air for a short period) as a response to temperature increase.

Differently from what has been observed for pollen of *Taxus* [9]—which represents a woody species highly sensible to temperature increase—by considering the general trend of the families, we can conclude that the duration of the pollen season of Betulaceae and Poaceae was not unequivocally altered by warming climate. Deepening the role of different trees in the Birch family, significant differences emerged by distinguishing the winter and spring blooming species: they actually are sensible to the temperatures of the season when microsporogenis starts.

Most evident consequences to climate change by reducing the release of pollen in the air are those in *Alnus*, and especially in *Corylus*. This study shows that winter-blooming tree species can be phenologically affected by global warming as this phenomenon has caused the increase of autumn temperatures. These trees react by end of the season earlier, and in general by shortening their pollen season. Surprisingly, this occurs in two widespread tree/shrub meso-hygrophilous genera – *Alnus* and *Corylus* – that therefore result less warmth-tolerance than other plants such as birch. This is partly in contrast with Central European models built by gridding meteorological/pollen data and by using them as predictor variables to build a model of high *Alnus*, *Corylus*, and *Betula* pollen concentration levels for spatially continuous areas of 11 cities in Poland [42]. In that research, the maximum range of the *Corylus* pollen season was 6–150 days of the year, 14–145 days for *Alnus*, and 35–164 days for *Betula* (calculated using the 99% method). The model predicts high pollen concentrations for the three genera, only suggesting a proportionally reduced increase of *Corylus*. Actually, the examination of data from pollen monitoring stations located in the same nation [43] suggests that hazel and alder pollination and the occurrence of airborne pollen vary greatly and are significantly influenced by meteorological conditions. The research introduces several variables, such as presence of locations exposed or sheltered to sun and wind, that have important influence on the pollen season of hazels and alders: for example, a delay in pollination start was observed in quite sunny but very windy sites.

Our data refer to mean data from a long series of aerobiological monitoring. Results support that the trends of pollen concentrations have some relationship with variations in temperatures. Probably these results especially reflect local evidence but they also mirror regional responses of plants to climate change. The phenology of *Alnus* and *Corylus* seems particularly affected by current global warming, and more than other Betulaceae trees and grasses. These trees have to be particularly taken into account as possible markers of plant responses to climate change.
